# Revalorisation of Sage (*Salvia lavandulifolia* Vahl) By-Product Extracts as a Source of Polyphenol Antioxidants for Novel Jelly Candies

**DOI:** 10.3390/antiox12010159

**Published:** 2023-01-10

**Authors:** Cristina Cedeño-Pinos, Magdalena Martínez-Tomé, María José Jordán, Sancho Bañón

**Affiliations:** 1 Department of Food Technology and Science and Nutrition, Veterinary Faculty, Regional Campus of International Excellence “Campus Mare Nostrum”, University of Murcia, 30100 Murcia, Spain; 2 CIBER: CB12/03/30038 Pathophysiology of Obesity and Nutrition, CIBERobn, Carlos III Health Institute (ISCIII), 28013 Madrid, Spain; 3 Research Group on Rainfed Crops for the Rural Development, Murcian Institute for Agricultural and Environmental Research and Development (IMIDA), 30150 Murcia, Spain

**Keywords:** candy, sage, antioxidants, polyphenols, salvianic acid, rosmarinic acid

## Abstract

Sage (*Salvia lavandulifolia* Vahl) aqueous extracts (SE) obtained from distillation by-products were assessed as antioxidants for nutritionally enhanced jelly candies. Two experimental SEs with a different content of phenolic acids and flavonoids were tested: (i) SE38 (37.6 mg/g) and (ii) SE70 (69.8 mg/g), with salvianic and rosmarinic acids as main polyphenols, respectively. Flavour alteration, stability of sage polyphenols, physical quality traits and antioxidant capacity (AC) were studied in strawberry candies formulated without sugars and enriched with SEs at 0.25, 0.50 and 0.75 g/kg. Despite their different quantitative composition, SE38 and SE70 provided similar antioxidant properties, which were dose dependent. Salvianic and rosmarinic acids were stable without degrading to candy processing (up to 80 °C), keeping their antioxidant potential. There were no relevant differences in flavour or physical traits (pH, °Brix and CIELab colour) between untreated and SE-enriched strawberry candies. The addition of 0.75 g SE/kg resulted in relevant increases of candy AC: (i) from 30 to 38 mg GAE/100 g (total phenolics); (ii) from 10 to 17 mg TE/100 g (DPPH^•^ radical scavenging assay); (iii) from 5 to 13 mg TE/100 g (ABTS^·+^ radical scavenging assay); (iv) from 84 to 163 µmol Fe^2+^/100 g (FRAP capacity) and (v) from to 75 to 83% (inhibition of deoxyribose damage). Sage distillation by-products can be revalorised as a source of natural antioxidants to produce healthier candies.

## 1. Introduction

The enrichment of food products with phenolic antioxidants is performed for a technological (stabilisation against oxidation) and/or nutritional purpose (intake of potentially functional compounds). Among the available sources of phenol compounds, Aromatic-Medicinal Plants (AMPs) are increasingly investigated for different food applications, particularly in products of low nutritional value such as candies [1], which are also naturally poor in antioxidants. Several AMPs, such as sage, rosemary or lavender, are cropped and subsequently distilled to produce essential oils for pharmaceuticals, dyes, biocides and cosmetics [2,3,4,5]. This generates oil-free by-products containing the polyphenol compounds not extracted with water steam, which can be used as natural, novel and safe antioxidants for the food industry, which contribute to revalorising these AMP crops [6]. Among the AMPs, sage is perhaps the largest genus of the *Lamiaceae* family, with more than 900 spices [7,8]. Interest in several sage spices focuses on their chemical composition, biological properties and industrial use of essential oil [3,8], and, most recently, on the oil-free sage extracts (SE) containing phenolic acids (e.g., salvianic and rosmarinic acids) and flavonoids (luteolin-7-*O*-glucoronide and luteolin-4-glucoside) [5,6]. Some research on the antioxidant properties in vitro of sage essential oil and extracts have determined that both can act as free radical scavengers, even equal or better than synthetic antioxidants, such as butylated hydroxytoluene [9,10,11]. However, sage essential oils are strongly aromatic and may alter flavour, limiting their possible use in food, while SEs may have more possibilities for use in sensorially compatible food products.

The US Food and Drug Administration (FDA) [12] currently recognises the essential oils, oleoresins (without solvents) and natural extracts (including distillates) from some sage species as safe, including *S. officinalis*, *fruticosa*, *sclarea*, and *lavandulifolia*. Similarly, the European Union (EU 2470/2017) [13] authorizes the use of chia (*S. hispanica*) oil and its whole or crushed seeds for human consumption. A handicap of using SEs as food antioxidants is the variability of their polyphenol content due to genetic, environmental and agronomic factors, among others, making it essential to standardize the plant material and extraction procedures used to obtain homogeneous extracts with suitable antioxidant properties [14]. In this regard, Herraiz-Peñalver et al. (2010) [15] carried out an extensive prospection (221 populations) of Iberian *S. lavandulifolia* varieties, selecting eight of these populations according to their yield and phenol composition for producing antioxidants. These researchers determined that sage with high polyphenol content showed higher radical scavenging activities, while that with lower polyphenol content showed remarkable iron and copper chelating activities, which could be of interest in the prevention of the oxidation process [6]. This reveals the importance of applying quantitative and qualitative criteria to select the starting material of sage to produce natural antioxidants for food. The antioxidant properties of SEs should be assessed with different assays, as these may exert antioxidant activities through several mechanisms, including ferrous iron chelation, peroxide decomposition and free radical scavenging [16].

Various SE have already been tested as food antioxidants in meat products [17,18,19,20] and goat milk-based beverages [21], though their use in candy products has been barely explored. Among these, jelly candies are consumed worldwide by both adults and children, perhaps due to their attractive chewy textures and fruity flavours. The global market of candies was valued at 2520 US$ millions in 2020 and a compound annual growth rate of 2.9% is expected for the 2022–2027 period [22]. From a nutritional point of view, jelly candies are considered unhealthy sugary products, poor in antioxidants; therefore, the development of healthier formulations presents great challenges. Current strategies are based on sugar replacement by sweeteners and/or dietary fiber [1,23,24,25,26]. In addition, some trials with plant extracts have been successfully conducted to obtain healthier candies reinforced with antioxidants from rosemary [1]; guajava leaf [27]; hibiscus [28]; white tea [29]; ginger [30]; peppermint [31]; propolis [32,33]; chokeberry [34] and dandelion leaf [35]. The antioxidant and/or sensory properties of enriched candies were studied in these trials. In jelly candies, rosemary and propolis extracts showed good antioxidant properties but also provided off-flavours, limiting their use as candy ingredients [1,33]. The degradation of added polyphenols is another relevant aspect for candy enrichment. It has been reported that rosmarinic acid, perhaps the most abundant polyphenol present in aqueous from rosemary and sage [36,37], may resist the cooking procedure conducted to obtain jelly candies [1]. Therefore, SEs might present some suitable properties for enriching candies: (i) SEs are not particularly astringent or bitter and have a mild chamomile flavour that might be more compatible with fruity aromas; (ii) their most abundant polyphenols (rosmarinic and salvianic acids) are water soluble and might be heat resistant and (iii) SEs are widely available, as they come from by-products of the distillation industry. Several studies provide evidence on the versatility of the jelly candy matrix, which allows the retention of bioactive compounds, either as preservatives, functional ingredients or nutraceuticals, possibly due to the triple helix structure of gelatine, which can present cross-linking and formation of three-dimensional networks [38,39,40]. The research hypothesis was that SEs may improve the antioxidant properties of enriched jelly candies without any detriment to sensory quality.

The objective was to assess the polyphenol profile and the antioxidant properties of two aqueous extracts obtained from two selected sage (*S. lavandulifolia*) ecotypes (with different quantitative polyphenolic profile) in novel jelly candies formulated with dietary fibre and sweeteners.

## 2. Materials and Methods

### 2.1. Experimental Design

A randomised factorial design with two experimental SEs (SE38 and SE70; 38 and 70 mg polyphenol/g extract) at four addition levels (0, 0.25, 0.50 and 0.75 mg/kg raw candy) was performed. The AC was previously validated in SE samples at the same concentrations used in candies. Three batches of candies were manufactured for each formulation (two SE × four addition levels). Different properties related with SE addition (flavour, phenolic acids stability, pH, soluble solids, colour and AC) were studied in jelly candies.

### 2.2. Sage Extracts

Two SEs from selected sage plants were manufactured in the laboratory of the Rain Feed Crops for the Rural Development Department part of the Institute for Agrifood and Environmental Research and Development, Murcia, Spain (IMIDA). Essential oil was previously removed from the sage leaves by distillation with water steam, according to the method described by Jordán et al. (2013) [41]. For the SEs, oil-free dry leaves were ground to 2 mm to obtain a powder. Sage powder was mixed with distilled water at 1:10 (*w*:*v*) ratio, kept at 30 °C for 90 min in a water bath with constant stirring and centrifuged at 4560× *g* and 5 °C for 10 min in a Digecen 21 R centrifuge (Orto Alresa, Madrid, Spain). The supernatant was filtered on Whatman filter paper (No. 4), lyophilised (Lyobeta 15, Telstar) at 100 mbar and −80 °C for 24 h. The dry SEs were packed into a dark steel container with nitrogen and kept at −80 °C and in darkness until further use.

### 2.3. Determination of Sage Polyphenols in Extracts and Candy Samples

SE polyphenols were determined according to methodology used in previous trials [41,42]. Dry extracts were dissolved in methanol and filtered (0.45 µm), and 20 µL extract (for candy samples, see subsection) were injected into an HPLC-1200 Series (Agilent, Waldbronn, Germany) equipped with a G1311A binary pump and a G1315A UV/Vis photodiode array detector. Flow rate was 1.0 mL/min, and detection wavelengths were set between 280 and 330 nm. Identification was performed by comparing retention times with the respective spectra, and quantification was carried out with linear regression models of standard dilutions. Standards were provided by (i) Sigma-Aldrich (St. Louis, MO, USA); Luteolin-7-*O*-glucoronide, Cirsimaritin, Hesperidin, Luteolin-4-*O*-glucoside, Lytospermic Acid, Salvianolic Acid, Protocatechuic Acid and Cirsileneol; by (ii) Acros Organics (Janssen-Pharmaceuticalaan, Geel, Belgium); Caffeic Acid; by (iii) Fluorochem (Hadfield, Glosop, UK); Salvianic Acid; by (iv) Fluka (Fisher Scientific, Madrid, Spain); Rosmarinic acid; and by (v) Biosynth (Carbosynth, Compton, UK); Salvigenin. Results were expressed as mg/g extract or µg/g candy.

### 2.4. Jelly Candy Manufacturing

Inulin cream and gelatine solution were prepared beforehand. Inulin and fructooligosaccharides (FOS) syrup were mixed in a Ultraturrax (11,500 rpm; 25 °C; 5 min) until a cream was obtained. Pork gelatine was dissolved in hot water (2:1 *w*/*w*) (80 °C; 30 min) with constant stirring. Cream (inulin + FOS syrup) and gelatine solution were then mixed (80 °C; 10 min) in a Mycook food processor (Taurus, Lérida, Spain). To the above mixture, the remaining ingredients (acids, flavouring, colouring and SE) were previously dissolved in water and then homogenised (80 °C; 5 min). Total content of soluble solids of the hot liquor was adjusted with water to 78 ± 0.1 °Brix (°B) (g/100 g), measured with a hand-held refractometer (Atago, Tokyo, Japan) Ingredients and quantities are shown in Table 1. The hot liquor was poured into pre-conditioned (30 °C; 10% RH; 24 h) starch powder moulds (printed on trays). Trays with hot candies were kept in a Climacell 707 climatic chamber (MMM Medcenter Einrichtun-gen, Munchen, Germany) with air circulation (25 °C; 30% RH; 24 h). After drying, candies were unmoulded, treated with carnauba wax and stored in polypropylene bags and in darkness (25 °C; 3 days) until analysis. The average final weight of candy units was 2.2 ± 0.2 g.

### 2.5. Sensory Analysis

Eight experienced panellists from the university staff were selected for the sensory trials to assess if SE affected candy flavour. Panellists received additional specific training. Three sessions were conducted where different water solutions and candies prepared with SE at 0–1 mg/g were studied to identify sage off-flavour. It was considered that a threshold concentration of 0.75 g SE/kg raw candy might modify candy flavour. A triangular test [43] was carried out where the flavour of untreated vs. SE38 or SE70 candies (at 0.75 g/kg) was compared. Samples were coded and presented in random order. Three samples, two identical, were presented. Panellists selected the sample which they considered different (forced judgement). A total of twenty-eight tests (four per panellist) were performed for the candies. The statistical significance of the number of correct vs. total number of answers was then determined.

### 2.6. Physical Assessment 

Moisture content (g/100 g) was determined by dehydration in a D6450 oven (Heraeus, Madrid, Spain) (100 °C; 24 h) (AOAC, 2000). Total content of soluble solids (g/100 g) was measured in thinly laminated samples with an Atago hand-held refractometer. CIELab colour was measured by reflectance on the surface of the caramel using a Chroma Meter II CR-200/08 (Minolta Ltd., Milton Keynes, UK) with a D65 illumination standard, a 2° observation angle and an aperture size of 50 mm. Results were expressed in CIE (Commission Internationale de L’éclairage) units: lightness (L*), redness (a*) and yellowness (b*). For the pH, 1 g candy was dissolved in 10 mL water (60 °C) and measured at 25 °C with a Crison 2001 pH meter (Barcelona, Spain).

### 2.7. Sample Extracts for Polyphenol Recovery and Antioxidant Capacity

Methanolic solutions were used to determine the AC (including total phenolics) and concentrations of rosmarinic and salvianic acids in candy and SE samples. 3 g candy was melted at 60 °C and dissolved in methanol to 10 mL using a calibration flask. The diluted sample was shaken (25 °C; 10 min) and then centrifuged (2580× *g*; 10 min; 25 °C) in a D2010 centrifuge (Kubota, Tokyo, Japan). The supernatant was collected in an Eppendorf tube and centrifuged again (6596× *g*: 10 min) using a D-37520 centrifuge (Biofuge Pico, Heraeus, Germany). Finally, the supernatant was stored at −80 °C until analysis. SE samples were directly dissolved in methanol at the same concentrations used as with candies.

### 2.8. Total Phenolic Content (TPC) 

TPC was determined using a modified version of the Folin–Ciocalteu method [44]. In a 10 mL volumetric flask, 5 mL distilled water, 250 µL sample and 800 µL Folin–Ciocalteu reagent were added. After 8 min, 1.2 mL aqueous sodium carbonate solution (1:5 *w*/*v*) was added to the above mixture and kept in a water bath (20 °C; 2 h). Absorbance was measured at 760 nm using a UV-VIS spectrophotometer (Genesis 180, Madison, WI, USA). Gallic acid (GA) (Merck KGaA, Darmstadt, Germany) was used as standard for quantification, using a calibration line (R^2^ = 0.999) at concentrations 0.1–6 µg GA/mL. Results were expressed as mg gallic acid equivalents (GAE)/100 g candy.

### 2.9. 2,20-Azino-bis-(3-ethylbenzothiazoline-6-sulfonic Acid) (ABTS•) Assay

ABTS radical cation decolourisation was determined according to Re et al., (1999) [45]. ABTS and Trolox (±-6-hydroxy-2,5,7,8-tetramethylchromane-2-carboxylic acid) reagents were purchased from Sigma Aldrich (Merck, Darmstadt, Germany). 10 mL aqueous solution of ABTS (7 mM) was prepared. The ABTS+ radical cation was formed by reaction of ABTS with 1 mL potassium persulfate (2.45 mM). The mixture was first kept in darkness (25 °C; 16 h) and absorbance (734 nm) was then adjusted to 0.7 using distilled water. 15 µL sample mixture and 985 µL ABTS solution was mixed, incubated in darkness (6 min), and absorbance (734 nm) was then measured. Quantification was performed using a calibration line (R^2^ = 0.9997) at concentrations of 0.1–5 µg Trolox/mL. Results were expressed as mg Trolox equivalent (TE)/100 g candy.

### 2.10. 2,2-Diphenyl-1 picrylhydrazyl (DPPH) Assay

DPPH radical scavenging activity was determined according to Brand-Williams et al. (1995) [46]. 3.5 mg DPPH reagent (Sigma-Aldrich) was dissolved in 10 mL methanol and kept in darkness (30 min). Absorbance (517 nm) of the DPPH solution was adjusted to 1.0 with methanol. 15 µL sample extract and 985 µL DPPH solution were mixed, incubated in darkness (10 min), and absorbance (517 nm) was then measured. A calibration line (R^2^ = 0.9991) was used for quantification at concentrations from 0.1 to 5.5 µg Trolox/mL. Results were expressed as mg Trolox equivalent (TE)/100 g candy.

### 2.11. Ferric Reducing Antioxidant Power (FRAP) Assay

FRAP capacity was determined according to Benzie and Strain, (1996) [47]. To prepare the FRAP reagent, 300 mM acetate buffer (pH 3.6); 10 mM 2,4,6-tripyridyl-triazine diluted in 40 mM HCl, and 20 mM FeCl_3_ 6H_2_O were mixed in a 10:1:1 ratio (*v*/*v*/*v*). 1.2 mL FRAP reagent and 40 µL sample were added and incubated in a water bath (37 °C; 2 min). Absorbance of the mixture was measured at 593 nm. A calibration line (R^2^ = 0.9991) was prepared at concentrations of 0.1 to 0.7 mM Fe^2+^ (FeSO_4_ × 7H_2_O). Results were expressed as µmol of Fe^2 +^ or FeSO_4_ equivalents per 100 g candy.

### 2.12. Percentage Inhibition of Deoxyribose Damage

The products of OH attack on deoxyribose were measured according to Martínez-Tomé et al., (2015) [48] with some modifications. The reaction was conducted with and without ascorbic acid to learn whether analytes act as primary antioxidants. 5 g candy was dissolved in 25 mL Mili-Q water (60 °C; 3 min) under constant stirring. The reaction mixture (final volume 1.2 mL) contained 100 µL ethylenediaminetetraacetic acid, 50 mM FeCl_3_, 2.8 mM deoxyribose, 2.8 mM H_2_O_2_, 50 µL sample and/or SE solutions, and 10 mM KH_2_PO_4_-KOH buffer (pH 7.4). Ascorbate (100 µM) was added to initiate the reaction, and tubes were incubated (37 °C; 1 h). Next, 1 mL 2-thiobarbituric acid and trichloroacetic acid were added and incubated (80 °C; 20 min). Finally, 2 mL butanol was added and centrifuged (2560× *g*; 20 min). Absorbance was measured at 532 nm and results were expressed as the percentage inhibition of deoxyribose attack, where 100% attack is defined as the absorbance level recorded for deoxyribose without the addition of tested compounds (control).

### 2.13. Statistical Analyses

Significance (*p* < 0.05) of the responses (*n* = 28) from the triangular sensory test was determined using probability tables (ISO, 4120:2004). The effects of SE addition on the dependent variables were determined using a one-way ANOVA (*n* = 72). The Tukey range test was used to compare group means (*p* < 0.05). Data were analysed with the Statistix 8.0 software for Windows (Analytical Software, Tallahassee, FL, USA). A discriminant analysis was performed to classify candies according to their overall AC using the Statgraphics 5.0 Plus package (Statpoint Technologies, Warrenton, VA, USA).

## 3. Results

### 3.1. Polyphenols and Antioxidant Capacity of Sage Extracts

Quantitative polyphenol profiles determined in both SE (mg/g extract) are shown in Table 2. Six phenolic acids (salvianic, protocatechuic, caffeic, salvianolic, rosmarinic and lithospermic) and six flavonoids (luteolin-4-*O*-glucoside, luteolin-7-*O*-glucoronide, cirsimaritin, hesperidin, salvigenin and cirsileneol) were quantified. As expected, both SEs share the same qualitative but not quantitative major profiles. Thus, SE38 recorded a total of 37.64 mg/g, with salvianic acid, luteolin-7-*O*-glucoronide and rosmarinic acid as the most abundant polyphenols, and for SE70 69.82 mg/g were reported with rosmarinic acid, luteolin-7-*O*-glucuronide and salvianic acid as most abundant polyphenols. SE70 contained more quantity of polyphenols than SE38, but in different proportions.

The AC of the SEs were checked at the same concentrations used in candies (Table 3). Overall, both SE showed dose-dependent antioxidant activities at the three doses tested. AC values obtained through the different assays were similar (TPC, DPPH and MR + DR) or even better at some concentrations (ABTS and FRAP) for SE38 than for SE70. TPC ranged similar for SE38 (13.13–27.74 mg GAE/100 g) and SE70 (13.25–26.95 mg GAE/100 g), therefore no relationship was found between the total amount of polyphenols quantified (HPLC-DAD) and that estimated in extracts with the Folin–Ciocalteu reaction. The ability to reduce the DPPH radical was also similar for SE38 (5.27–15.89 mg TE/100 g) and SE70 (4.25–14.59 mg TE/100 g), while SE38 (12.59–17.57 mg TE/100 g) showed higher scavenging capacity of ABTS at some concentrations than SE70 (8.65–15.06 mg TE/100 g). Similarly, the ferric ion reduction potential (FRAP) was also higher at some concentrations in the SE38 (74.20–164.20 μmol Fe^2+^/100 g) than in the SE70 (52.30–163.91 μmol Fe^2+^/100 g). In contrast, concentration and type of SE had fewer clear effects on the AC, assessed as inhibition percentage of deoxyribose damage (MR + DR). MR + DR percentages ranged similar in SE38 (20.07–25.54%) and SE70 (22–61—27.86%); when ascorbic acid was omitted from the reaction, the absorbances of both SEs were lower than Control, thus, sage antioxidants can be considered primary antioxidants, capable of attacking OH radicals. Despite SE38 containing about half of total polyphenols it showed similar AC than SE70 at the concentrations used in candies.

### 3.2. Effects of Sage Extracts on Jelly Candies

**Figure 1:**
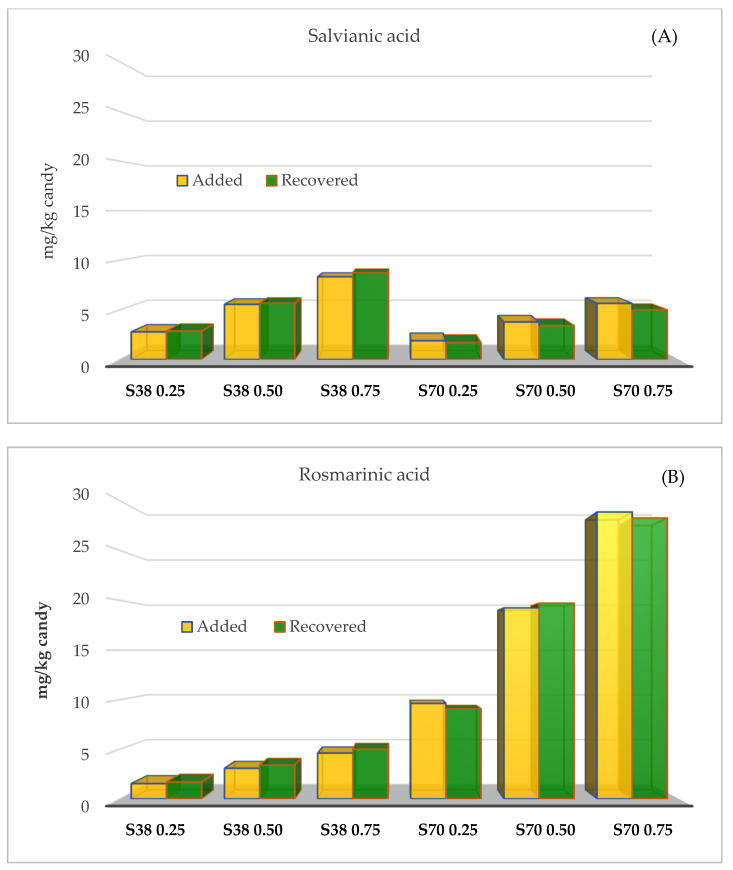
Remaining concentrations (mg/kg) of salvianic (**A**) and rosmarinic acid (**B**) determined in jelly candies enriched with sage extracts (SE70 and SE38) at different concentrations.

The concentrations of salvianic and rosmarinic acids determined in jelly candies (mg/kg candy) were similar to those used for candy formulation. Raw candies were formulated with 2.8–8.3 (SE38) and 1.9–5.7 mg/kg (SE70) of salvianic acid, while concentrations of this acid in the final product were 2.9–8.7 (S38) and 1.9–5.9 mg/kg (SE70) (Figure 1A). Raw candies were formulated with 1.5–4.6 (SE38) and 9.6–28.9 mg/kg (SE70) of rosmarinic acid, while concentrations of this acid in the final product were 1.7–5.0 (SE38) and 9.1–28.3 mg/kg (SE70) (Figure 1B). Therefore, most of the added salvianic and rosmarinic acids remained in the final product without degrading.

**Figure 2:**
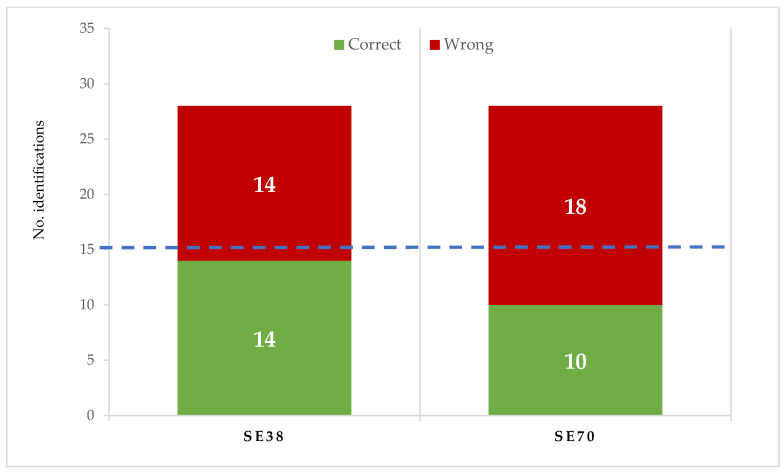
Identification of jelly candy flavour in the triangle test made with untreated and enriched samples (at 0.75 g/kg of SE70 or SE38). 15 or more correct identifications are required to be significant (*p* < 0.05) (ISO 4120:2004).

The contribution of both SEs to candy flavour was checked. In training sessions, SE flavour was described by the trained tasters as mild camomile and herbal, without bitterness or astringency. Both SEs showed a similar flavour to SE38. When compared with untreated candies in the triangular test (Figure 2), tasters correctly identified the flavour in 10 of 28 SE70 and 14 of 28 SE38 candy samples, respectively. According to the ISO Norm, 4120: 2004 [43], at least 15 of 28 correct identifications are required to determine that flavour differences are significant for *p* < 0.05; therefore, no relevant differences in flavour were found between untreated and SE-enriched strawberry candies.

Physical assessment is shown in Table 4. The addition of either SE38 or SE70 at any dose did not affect the pH value, moisture content and total soluble solid content of candies. Candies generally presented pH values around 3.1, solid soluble total content about 85 °B and moisture content ranging from 19–20 g/100 g. The moisture content formulated was 23.8 g/100 g (contained in FOS syrup 72 °B and water); therefore, jelly candies lost some quantity of water after processing. CIELab colour was also similar in candies from any formulation, except for those prepared with 0.75 g SE70/kg, which present lower L* than the rest, showing some incipient darkness. Thus, the addition of both SEs at the doses tested had no relevant influence on physical traits such as acidity or colour in jelly candies containing citric, lactic and carminic acids.

The values of AC determined in jelly candies are shown in Table 5. Overall, the SE38 and SE70 candies showed higher AC values for TPC, DPPH and FRAP than those untreated, and, to a lesser extent, for ABTS and MR + DR. Both SEs also showed dose-dependent antioxidant activities in candies. TPC (mg GAE/100 g) values were higher in the SE38 (up to 37.79) and SE70 (up to 36.06) candies than in the untreated candy (28.52); SE38 provided better results for TPC than SE70 in candies at 0.50 and 0.75 g/kg. The values of DPPH radical scavenging capacity (mg TE/100 g) were much higher in the SE38 (up to 16.78) and SE70 (16.75) candies than in the untreated candy (5.92); in this case, SE38 and SE70 provided similar results for DPPH in candies. Similarly, the values of ABTS radical scavenging capacity (mg TE/100 g) were much higher in the SE38 (up to 12.63) and SE70 (up to 13.15) candies than in the untreated candy (4.85), although the use of either SE at 0.25 g/kg did not improve ABTS values. The values of FRAP (μmol Fe^2+^/100 g) were also much higher in the SE38 (up to 162.52) and SE70 (up to 160.99) candies than in the untreated candy (51.37); unlike those seen for other AC assays, FRAP values were slightly higher in the SE70 than in the SE38 candies at 0.50 g/kg. The values of MD + DR hardly discriminated AC among formulations. The inhibition percentage (%) of deoxyribose damage determined in untreated candies (74.92) only improved in those containing 0.75 g/kg of either SE38 (77.79) or SE70 (82.59). Likewise, when ascorbic acid was omitted from the reaction, absorbances (532 nm)) were lower for SE candies (0.16–0.18) than in the untreated candy (0.22) and Control+ values (0.31), confirming that jelly candies acted as primary antioxidants scavenging OH· radicals generated during the reaction. With some exceptions, the antioxidant effects reproduced in extracts and candies show evidence of the antioxidant potential of sage polyphenols.

**Figure 3:**
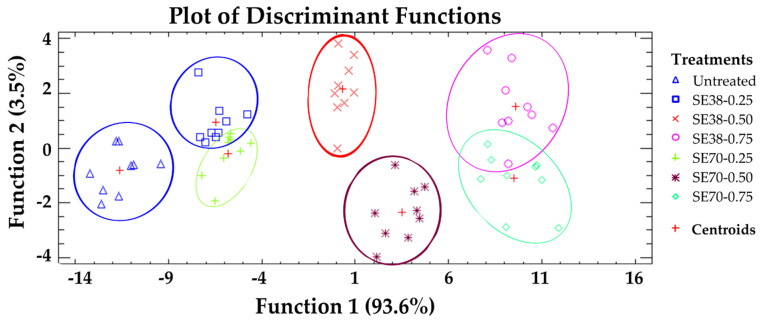
Discriminant analysis: classification of jelly candies according to their overall antioxidant capacity.

A discriminant analysis was performed to classify jelly candies according to their overall AC (Figure 3). The projections generated allowed a more intuitive identification of the different treatments. Five statistically significant discriminant functions were obtained (*p* < 0.05), with functions 1 and 2 explaining 97.1% of total variance. A clear separation was observed among untreated and SE-enriched candies, indicating the strong influence of SE on the AC values described above. A high overlap was seen between candies formulated with 0.25 and 0.75 g SE/kg, while those formulated with 0.50 g SE/kg showed a slight separation between the centroids. Thus, SE concentration played a more relevant role for candy AC than the SE used, confirming that both SEs provided similar antioxidant properties, despite their different composition.

## 4. Discussion

Plant extracts are often tested as antioxidants for food applications without making a previous typification of their composition and antioxidant properties. Natural extracts from plants contain different active compounds (phenolics and others) with different antioxidant activities and synergies, with it being difficult to know the exact antioxidant role of each compound. SE38 and SE70 came from different sage ecotypes selected according to their polyphenol content, with a respective predominance of rosmarinic acid. As seen, the major polyphenols present in both SEs were the same, though at different quantities and proportions. This was also the case when comparing different residues from high oleaginous-yielding *S. lavandulifolia* plants [6]. The total content of polyphenols reported by these authors ranged from 39 to 96 mg/g extract, with salvigenin and rosmarinic acid as major polyphenols in most populations. Our results agree with the total polyphenol content and rosmarinic acid predominance, but not for levels of salvigenin, which were very low (0.2 mg/g) in SE38 and SE70. Rosmarinic acid, an ester of caffeic and salvianic acids, is considered one of the most common polyphenols in the *Lamiaceae* family [49,50]. In fact, several studies confirm solid residues of different sage species to be rich in this acid [6,36,51,52,53]. There is little information on the presence of salvianic acid in SE. This compound, also known as ¨Danshensu¨, is one of the main antioxidants present in the aqueous extracts of the dried root of *Salvia miltiorrhiza* [16,54], a Chinese medicine used for treatment of cardiovascular disease, hepatitis, hepatocirrhosis, chronic renal failure and dysmenorrhoea [55]. Salvianic acid was also quantified (1.6–16 mg/g extract) in rosemary aqueous extracts containing 74–146 mg/g of total polyphenols that were used to enrich jelly candies [1].

The determination of AC in SE samples at concentrations tested in candies revealed the following: (i) the antioxidant activity provided by each SE was dose-dependent for all tested assays; and (ii) SE38 polyphenol extract had better antioxidant properties than SE70 when tested against the ABTS+ and the FRAP tests, but not for the DPPH radical scavenging activity. Differences found among the DPPH and the other two in vitro antioxidant tests are directly related to the chemical principles upon which they are based. This highlights the importance of reporting more than one in vitro antioxidant test, especially when the main objective is to know the possible health-promoting activity in vivo of these SEs [56]. It is also noteworthy that, attending to these results, in Spanish sage extracts, rosmarinic acid is not the most potent antioxidant compound. To assert this, by considering the SE38 polyphenolic profile, higher contents of salvianic, caffeic and salvianolic acids were found. These polyphenols are deemed to have high antioxidant power [16]; salvianic acid may actually act as a secondary antioxidant, being degraded to protect other compounds prone to oxidation. Bioactivity of phenolic antioxidant mainly depends on the combination of aromatic rings and OH groups that (re)assemble their chemical structure in order to bind and prevent oxidation [57]. The antioxidant activity of sage polyphenols (including caffeic acid and its derivatives) would depend not only on the configuration of these molecules, but also on the number and position of the OH group [58,59]. Whatever the case, the antioxidant activities of SEs are the result of a pull of different active compounds that are simultaneously acting in the candy matrix. AC results obtained for SE38 and SE70 are coherent with those found in other SEs through different assays (DPPH, ABTS, FRAP and others), which also had good antioxidant properties [6,19,52,60]. These authors suggest that plant extracts rich in phenolic acids and flavonoids tend to have higher AC [51,61].

Stability of sage antioxidants is a crucial aspect for development of enriched candies. The jelly candy matrix favours the retention of bioactive compounds, including antioxidants, in the three-dimensional networks formed by gelatine [38]. As seen, salvianic and rosmarinic acids resisted quite well the processing conditions (temperature, oxygen exposure, acidity, etc.) conducted to obtain jelly candies. Rosmarinic acid stability was already checked in similar jelly candies enriched with rosemary extracts, where this acid was largely the most abundant polyphenol (35–77 mg/g extract) [1]. Thermal resistance of rosmarinic acid to cooking processes has also been reported for other food products, such as baked cookies (190 °C; 10 min) enriched with rosmarinic acid [62] and baked bread (230 °C; 23 min) enriched with aromatic plant extracts [63]. However, to our knowledge, there are no studies that analyse salvianic acid in food after cooking or storage, which hinders contrast with our results. Nevertheless, both salvianic and rosmarinic acid remained practically without degradation in the final product and it can be assumed they (and probably other sage polyphenols) can act as antioxidants during candy shelf life, contributing to oxidative stability and/or functional properties. Stability of bioactive compounds is crucial in developing food with functional benefits. The intake of microquantities of sage polyphenols (up to 52 mg/kg) through the consumption of jelly candies does not initially appear relevant in a diet rich in fruits and vegetables, where the intake of polyphenols can reach 2.6–3.0 g/person/day [64], although it could contribute to improving the diet of candy consumers, particularly children and teenagers. Sensory changes resulting from the addition of new ingredients to foodstuffs are important, as these can influence consumer acceptance and purchasing decisions. As mentioned, sage extracts may provide herbal off-flavour to candies. In the present study, the idea was to develop enriched candies free of sage off-flavours, destined to common consumers. Sensory analysis conducted with trained tasters found that adding 0.75 g/kg (SE38 or SE70) did not alter the flavour of strawberry candies. In previous studies with enriched jelly candies (with rosemary or propolis extracts), consumers more than trained tasters had more difficulty discriminating herbal flavours, meaning a negative result (flavour identification) from a triangular test with experts can be extrapolated to consumers [1,33]. In these studies, alteration of candy flavour due to rosemary off-flavours (described as herbal, astringent and bitter) were detected by trained tasters at 0.26 g extract/kg raw candy, but not by consumers. Similarly, consumers began to perceive propolis off-flavours (described as bitter, pungent and astringent) at 0.20 g extract/kg raw candy, a concentration clearly detected by trained tasters. There is a high disparity in doses from plant extracts used in other studies, which could be attributed to their different origin, concentration and composition, and whether plant extracts are used or not as flavouring agents for candies. Available studies, including ours, focus on candy acceptability but not on flavour alteration. For example, lemon candies enriched with tea extracts were well accepted by consumers when using 10–15 g dry extract/kg candy [65], whereas the acceptance of candies with dandelion extracts decreased with increasing dosage from 5 to 10 g dry extract/kg candy, which was attributed to the bitterness and astringency of this extract [35]. Bitterness is related to the flavonoid fraction in tea extracts [66], while camomile flavour is related with the presence of some volatiles from sage residues (e.g., 1-Borneol, 1–8-Cineole, p-Cymene, Linalool, spatulenol or t-Anetol) [67]. SEs would have a more tolerable flavour than rosemary or propolis extracts when used in strawberry jelly candies.

Physical measurements such as pH, total solids and CIELab colour are often conducted in factories to assess candy quality. Jelly candy is a product based on carbohydrates solubilized in water, strongly acidified, whose moisture content was adjusted at the end of cooking; therefore, it is unlikely that the addition of micro quantities (up to 0.75 g/kg) of SE would result in relevant changes of pH, soluble solid content or moisture content. As observed, the pH values (over 3.15), total soluble solids (over 85 °B) and moisture content (over 19 g/100 g) were similar in untreated and enriched candies, despite SE-content acids and other water-soluble compounds. Neither did the addition of extracts from rosemary or propolis modify the pH value of jelly candies in other studies [1,33]. SE38 and SE70 are brown powders that may interfere with candy colour. The chromatic coordinates of the candies coloured with micro quantities (0.5 g/kg) of carminic acid were not affected by SE addition, although a slight darkness was found in candies with 0.75 g SE70/kg (but not with SE38). To the naked eye, SE70 powder was slightly darker than SE38, suggesting it might be richer in chlorophyll derivates and other pigments. A spectrophotometric scanning (190–600 nm) of the equivalent methanol solutions at 0.75 g/kg confirmed that SE70 presented higher absorbances than SE38, in accordance with changes observed in candy lightness. In previous trials [1,33], changes observed in the CIELab colour of jelly candies with lower quantities (up to 0.25 mg/kg) of rosemary or propolis green-brown powders were less clear and were influenced by moisture variations. Similar trends have been observed in candies with guajava (Psidium guajava) leaf extract, where lightness decreased as extract concentration increased [27]. Likewise, Altınok et al. (2020) [68] stated that decrease in lightness in candies with grape powder might be caused by insoluble particles in suspension coming from the extract. In any case, it is unlikely that small changes in candy lightness can be detected in an appearance test.

SE38 provided similar or even better antioxidant properties than SE70 in candies, with some exceptions. AC results for candies depend on the chemical test used and the contribution of other candy components to the antioxidant status. By extrapolating results obtained from the quantification of the salvianic and rosmarinic acids in candies, it can be deduced that most sage polyphenols maintain intact their antioxidant potential in candy samples. Five different AC assays were tested for the experiment. In theory, the antioxidant effects of sage polyphenols on candies should be detected with more difficulty at low (0.25 g/kg) concentrations of extracts. As observed, 0.25 g/kg of either SE38 or SE70 were sufficient to improve candy AC assessed by TPC, DPPH and FRAP, but not ABTS and MR-DR, which required higher concentrations. Therefore, the relationship between AC value and SE concentration was more evident in some assays than in others, it being likely that other candy components might have interfered with results. TPC, DPPH and FRAP suitable methods to assess differences of AC in jelly candies enriched with sage polyphenols. The Folin–Ciocalteu method quantifies the antioxidant response of phenolic concentrations, though other compounds present in candy ingredients containing aromatic rings (carminic acid and strawberry aromatic esters), fructose residues (FOS and inulin) or free amino acids (pork gelatine) may present some positive response [1,69,70]. DPPH and FRAP are widely contrasted antioxidant assays for plant and food samples that contain phenolic antioxidants capable of donating a hydrogen, acting as radical scavengers. ABTS is based on the ability of antioxidants to extinguish the ABTS+ upon different reaction conditions, and their values partially depend on the number of free phenolic hydroxyls and type of linkage structures in the food matrix [71]. The deoxyribose assay detects possible OH· radical scavengers. Higher OH· radical scavenging was observed in SE-enriched candies than in SE, perhaps due to the synergistic effect of the different ingredients (e.g., citric acid, inulin and SE, among others) used for candy formulation. It should be noted that candies show a primary antioxidant behaviour by scavenging OH radicals, unlike other foods with antioxidant activity such as coffee, which acts as a secondary antioxidant [1,72].

The reactivity of polyphenols is also modulated by their concentration, as some can participate in more than one reaction (e.g., reducing a metal and donating an electron), causing them to act as prooxidants at low doses, and as antioxidants at high doses [73]. At SE doses which can be used in jelly candies without altering their flavour, polyphenol profile seems to be as or more important than the total concentration for the resulting antioxidant status. It is possible to formulate SE-enriched candies of similar AC by using SEs with different polyphenol profiles. However, little is still known regarding the possible antioxidant action mechanisms of the different sage compounds in candies and other food matrices. There is growing research for new natural sources compatible with the characteristics of candies to increase their antioxidant efficacy. In this context, Nguyen et al. (2022) [35] found increases of TPC to 39.8 mg GAE/100 g, and of the relative AC to 31.7% (DPPH) and 94.9% (ABTS) when dandelion extract was added to candies. Similarly, candies enriched with grape powder (wine by-product) rich in anthocyanins, flavonols and procyanidins increased their AC [74]. Likewise, Amjadi et al. (2018) [75] reported that betanin-enriched candies inhibited 55–87% of DPPH radicals.

## 5. Conclusions

Sage distillation by-products can be revalorised as a source of natural antioxidants for food applications. Jelly candies are suitable products to develop novel formulations enriched with sage polyphenols that increase antioxidant status. Analysis of the composition and antioxidant capacity provides evidence that the criteria for choosing sage extracts should be based on both the total content and the proportion of different phenolic acids and flavonoids. Sage extracts have good sensory properties, which favours candy enrichment with polyphenols. Compared to other similar extracts (e.g., rosemary and propolis), sage extracts contain a lower quantity of polyphenols but can be used at higher doses in jelly candies. Salvianic and rosmarinic acid, the most abundant polyphenols present in sage aqueous extracts, are resistant to the manufacturing conditions conducted to obtain candies and keep their antioxidant potential. The properties of sage antioxidants would be affected by the chemical interactions with other candy components, so that an increase in the dose of the extract may not provide an equivalent antioxidant enhancement in enriched candies. Nutritional quality of jelly candies can be improved by introducing microquantities of sage extracts rich in polyphenols, along with other strategies (e.g., sugar replacement). Genetic selection of sage plants with polyphenolic chemotypes without such a predominance of rosmarinic acid may improve the technological (oxidative stability and shelf life) and nutritional (functional properties through intake and toxicological implications) properties of enriched candies, questions that should be elucidated.

## Figures and Tables

**Table 1:** Ingredients of raw jelly candies.

Ingredients	g/kg	Supplier
Fructooligosaccharides (FOS)	705.8	Fosvitae chemical synthesis 72 °B, Zukán
Chicory inulin	114.4	Orafti GR Beneo, Barcelona, Spain
Water	110.8	
Pork gelatine type A	41.0	Juncà Gelatines, Barcelona, Spain
Citric acid	12.0	Helm Ibérica, Madrid, Spain
Lactic acid	6.0	Brenntag Chemistry, Sevilla, Spain
Strawberry flavouring	3.0	PIM, Scentium Flavours, Murcia, Spain
Stevia powder	1.4	Zukán, Murcia, Spain
Sodium citrate	1.0	Azelis, Barcelona, Spain
Carminic acid	0.5	Bright’n RED Carmine 50 WS, Marcovic, Slovenia
Sage extract (SE)	0.25, 0.50 or 0.75	IMIDA, Murcia, Spain

**Table 2:** Polyphenol content (mg/g extract) of the two sage aqueous extracts (SE38 and SE70).

Polyphenols	SE38		SE70	
	Mean	SEM	Mean	SEM
Phenolic acids				
Salvianic acid	11.11	0.121	7.54	0.054
Protocatechuic acid	0.23	0.053	0.27	0.032
Caffeic acid	3.81	0.040	2.24	0.070
Salvianolic acid A	1.19	0.081	0.99	0.051
Rosmarinic acid	6.15	0.163	38.48	0.153
Lytospermic acid	2.67	0.092	1.28	0.015
Flavonoids				
Luteolin-4-glucoside	3.55	0.051	3.14	0.024
Luteolin-7-O-glucoronide	7.62	0.090	13.14	0.113
Cirsimaritin	0.22	0.022	0.29	0.012
Hesperidin	0.98	0.257	2.01	0.286
Salvigenin	0.20	0.031	0.21	0.085
Cirsileneol	<LQ		0.24	0.036
Total content	37.64		69.82	

Abbreviations: SE38 and SE70: sage extract of different polyphenol content; SEM: Standard Error of Mean.

**Table 3:** Antioxidant activity assessed in sage extracts (SE38 and SE70) at the same concentrations used in jelly candies.

Sage Extracts	TPC	DPPH	ABTS	FRAP	MR + DR	MR + DR Omit ASC
	mg GAE/100 g	mg TE/100 g	mg TE/100 g	µmol Fe ^2+^/100 g	% Inhibition	A_532_
Control						0.31 ^a^
SE38 (g/kg)						
0.25	13.13 ^c^	5.27 ^c^	12.59 ^c^	74.20 ^d^	20.07 ^c^	0.18 ^b^
0.50	18.60 ^b^	11.65 ^b^	14.38 ^b^	136.39 ^b^	22.38 ^b,c^	0.17 ^b^
0.75	27.74 ^a^	15.89 ^a^	17.57 ^a^	164.20 ^a^	25.54 ^a,b^	0.16 ^b^
SE70 (g/kg)						
0.25	13.25 ^c^	4.25 ^c^	8.65 ^d^	52.30 ^e^	22.61 ^b,c^	0.21 ^b^
0.50	19.08 ^b^	10.86 ^b^	12.45 ^c^	124.77 ^c^	23.80 ^a,b,c^	0.21 ^b^
0.75	26.95 ^a^	14.59 ^a^	15.06 ^b^	163.91 ^a^	27.86 ^a^	0.19 ^b^
SEM	0.277	0.568	0.334	2.497	1.348	0.023

Abbreviations: SEM: Standard Error of Mean; TPC: Total Phenolic Content; GAE: Gallic Acid Equivalent; ABTS: 2.2′-azinobis-(3-ethylbenzothiazoline-6-sulfonic); DPPH: 2,2-diphenyl-1-picrylhydrazyl radical; TE: Trolox Equivalents; FRAP: Ferric-reducing antioxidative power; MR + DR: OH Damage to deoxyribose. ASC: ascorbate. ^a–e^ Means with different superscripts are different for *p* < 0.05 (Tukey Test).

**Table 4:** Physical assessment of jelly candies enriched with sage extracts (SE70 and SE38) at different concentrations.

	pH	Soluble Solids	Moisture	L*	a*	b*
		g/100 g	g/100 g	CIE Units	CIE Units	CIE Units
Untreated	3.16	85.76	19.79	36.34 ^a^	36.09	11.18
SE38 (g/kg)						
0.25	3.11	84.92	19.29	35.77 ^ab^	36.49	10.62
0.50	3.11	85.65	19.08	35.09 ^ab^	36.70	10.91
0.75	3.14	85.76	19.41	34.41 ^ab^	36.19	10.48
SE70 (g/kg)						
0.25	3.15	85.03	20.03	35.38 ^ab^	36.12	12.06
0.50	3.14	85.76	19.91	34.96 ^ab^	36.29	13.18
0.75	3.18	83.98	19.83	33.31 ^b^	36.00	11.29
SEM	0.028	0.915	0.428	0.802	1.430	0.996

Abbreviations: SEM: Standard Error of Mean; L*: lightness; a*: Redness; b*: yellowness; CIE: Commission Internationale de L’éclairage. ^a,b^ Means with different superscripts are different for *p* < 0.05 (Tukey Test).

**Table 5:** Antioxidant capacity assessed in 100 g jelly candies enriched with sage extracts (SE38 and SE70) at different concentrations.

Candies	TPC	DPPH	ABTS	FRAP	MR + DR	MR + DR Omit ASC
	mg GAE/100 g	mg TE/100 g	mg TE/100 g	µmol Fe ^2+^/100 g	% Inhibition	A_532 nm_
Control						0.31 ^a^
Untreated	28.52 ^f^	5.92 ^d^	4.85 ^d^	51.17 ^e^	74.92 ^c^	0.22 ^b^
SE38 (g/kg)						
0.25	31.06 ^e^	10.13 ^c^	5.41 ^d^	83.53 ^d^	74.65 ^c^	0.18 ^c^
0.50	33.85 ^c^	13.09 ^b^	7.11 ^c^	124.23 ^c^	76.92 ^b,c^	0.18 ^c^
0.75	37.79 ^a^	16.78 ^a^	12.63 ^a^	162.52 ^a^	77.79 ^b^	0.17 ^c^
SE70 (g/kg)						
0.25	30.42 ^e^	10.46 ^c^	5.84 ^d^	87.50 ^d^	76.43 ^b,c^	0.18 ^c^
0.50	32.60 ^d^	13.46 ^b^	11.05 ^b^	134.74 ^b^	77.38 ^b,c^	0.17 ^c^
0.75	36.06 ^b^	16.75 ^a^	13.15 ^a^	160.99 ^a^	82.59 ^a^	0.16 ^c^
SEM	0.371	0.612	0.382	3.103	0.920	0.009

Abbreviations: SEM: Standard Error of Mean; TPC: Total Phenolic Content; GAE: Gallic Acid Equivalent; ABTS: 2.2′-azinobis-(3-ethylbenzothiazoline-6-sulfonic); DPPH: 2,2-diphenyl-1-picrylhydrazyl radical; TE: Trolox Equivalents; FRAP: Ferric-reducing antioxidative power; MR + DR: Damage to deoxyribose. ASC: ascorbate. ^a–e^ Means with different superscripts are different for *p* < 0.05 (Tukey Test).

## Data Availability

Data are contained within the article.
